# DermoGAN: multi-task cycle generative adversarial networks for unsupervised automatic cell identification on *in-vivo* reflectance confocal microscopy images of the human epidermis

**DOI:** 10.1117/1.JBO.29.8.086003

**Published:** 2024-08-02

**Authors:** Imane Lboukili, Georgios Stamatas, Xavier Descombes

**Affiliations:** a Johnson & Johnson Santé Beauté France, Paris, France; b UCA, INRIA, I3S/CNRS, Sophia Antipolis, France

**Keywords:** cycle generative adversarial network, identification, keratinocytes, multi-task, reflectance confocal microscopy, segmentation

## Abstract

**Significance:**

Accurate identification of epidermal cells on reflectance confocal microscopy (RCM) images is important in the study of epidermal architecture and topology of both healthy and diseased skin. However, analysis of these images is currently done manually and therefore time-consuming and subject to human error and inter-expert interpretation. It is also hindered by low image quality due to noise and heterogeneity.

**Aim:**

We aimed to design an automated pipeline for the analysis of the epidermal structure from RCM images.

**Approach:**

Two attempts have been made at automatically localizing epidermal cells, called keratinocytes, on RCM images: the first is based on a rotationally symmetric error function mask, and the second on cell morphological features. Here, we propose a dual-task network to automatically identify keratinocytes on RCM images. Each task consists of a cycle generative adversarial network. The first task aims to translate real RCM images into binary images, thus learning the noise and texture model of RCM images, whereas the second task maps Gabor-filtered RCM images into binary images, learning the epidermal structure visible on RCM images. The combination of the two tasks allows one task to constrict the solution space of the other, thus improving overall results. We refine our cell identification by applying the pre-trained StarDist algorithm to detect star-convex shapes, thus closing any incomplete membranes and separating neighboring cells.

**Results:**

The results are evaluated both on simulated data and manually annotated real RCM data. Accuracy is measured using recall and precision metrics, which is summarized as the F1-score.

**Conclusions:**

We demonstrate that the proposed fully unsupervised method successfully identifies keratinocytes on RCM images of the epidermis, with an accuracy on par with experts’ cell identification, is not constrained by limited available annotated data, and can be extended to images acquired using various imaging techniques without retraining.

## 1 Introduction

Reflectance confocal microscopy (RCM) is a non-invasive *in vivo* imaging technique that allows for visualization of epidermal cells, called keratinocytes, at a cellular level in the epidermis and upper layers of the dermis (150 to 200  μm in depth, depending on body site^1^). It provides information on the geometry and topology of the skin, which are key elements in the skin barrier and health, thus helping in the study of infants’ and children’s skin maturation, adult skin aging, and photo-aging due to ultraviolet (UV) exposure. RCM can also be used to assess skin inflammatory diseases, e.g., psoriasis, allergic contact dermatitis, and skin cancer,^2^ providing information faster than traditional biopsies and potentially reducing the number of unneeded biopsies, by guiding them to delimit lesion borders and helping in disease diagnosis and monitoring.

Although automated methods have been developed to identify some lesions on RCM images,^3^^–^^7^ most of the analysis is performed manually, which is time-consuming and subject to inter and intra-expert interpretation.^8^ Hence, an automated method for keratinocyte identification on RCM images is needed, which would allow for a more reproducible, unbiased, and precise analysis. Unfortunately, image quality, heterogeneity, and low signal-to-noise ratio are a hurdle to automated methods’ development. Attempts at automating keratinocyte identification on RCM images have been made and were based on the identification of cell morphological features, e.g., membrane size,^9^^,^^10^ but are hindered by manual parametrization often different among datasets, image types, and epidermal layers. Deep learning methods could be an alternative solution to circumvent these problems.

Accurate automated cell identification on biomedical images with deep learning has been a growing research topic in computer vision but is hindered by the lack of labeled data on account of cost, time, and domain-specific skills. Unsupervised learning bypasses the labeled data scarcity problem by tapping into unlabeled data potential. One of the main developments in unsupervised learning research in recent years is the cycle generative adversarial networks (cycle-GANs)^11^ for unpaired image-to-image translation and is classically used for synthetic image generation and data augmentation.^12^^–^^15^

We propose a top-down, structure-aware, multi-task cycle-GAN architecture, which we have named DermoGAN, to automatically detect keratinocytes on RCM images. The multi-task model performs two parallel cycle-GANs, to denoise RCM images while highlighting membrane positions, and provides an incomplete cell identification, which is then refined and completed by a post-processing based on star-convex shape detection. The proposed architecture is fully unsupervised and thus not limited by training annotations, often the first limitation to the use of deep learning methods in the analysis of biomedical images. To our knowledge, this is the first use of cycle-GANs in a multi-task framework. In addition, while generally used for synthetic image generation and data augmentation, here, we employ the cycle-GAN algorithm as an image-denoiser and cell-identifier. Indeed, we change our perspective on the cell identification problem and move the data augmentation approach consisting of learning the noise model in RCM images to create synthetic images, which will then be used to augment our dataset to be used in other models, to a new approach consisting of learning the image *denoising* model. This change in perspective makes use of the cycle-consistency property of cycle-GANs.

We compare the proposed method with seven other approaches, namely, a supervised method based on a U-net architecture,^16^ a pre-trained StarDist^17^ applied to Gabor-filtered images, two unsupervised approaches based on a cycle-GAN with different inputs, a tailored pipeline based on the detection of membrane morphological features,^8^ the CellPose^18^ algorithm for cellular segmentation, and finally DermoGAN followed by postprocessing using CellPose.

We demonstrate that the presented DermoGAN architecture performs on par with expert manual identification of cells and outperforms the seven other tested automated methods in accuracy and execution computational time. We also explore the use of DermoGAN, without retraining, to images acquired using other image acquisition techniques, and the possibility of training it on datasets made entirely of synthetic images.

## 2 Methods

### 2.1 Identifying Keratinocytes on RCM Images with DermoGAN

**Fig. 1:**
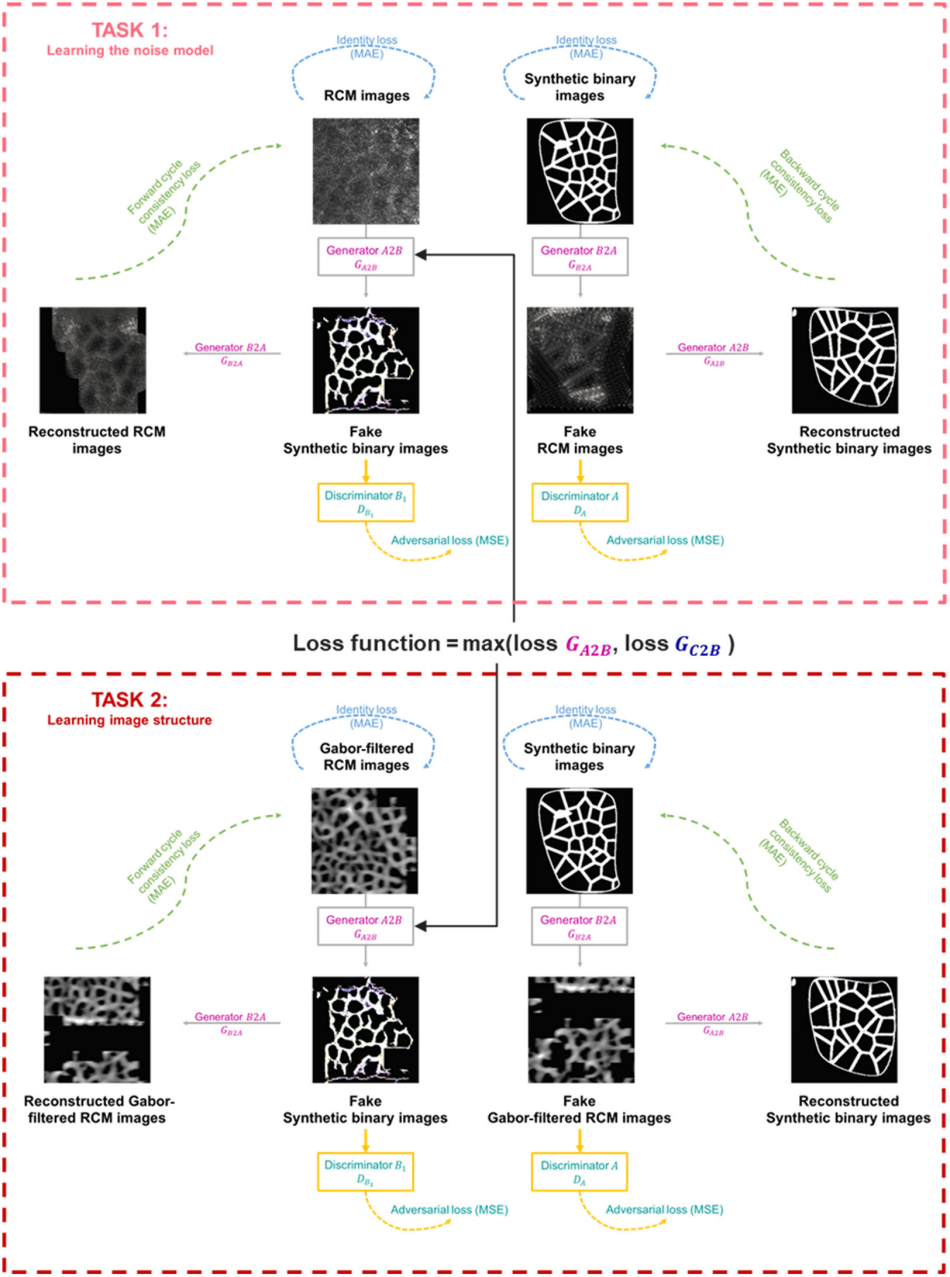
DermoGAN architecture. The first task maps RCM images to the unpaired synthetic binary images, whereas the second task learns the structure of RCM images of the epidermis by translating Gabor-filtered RCM images into binary images.

The goal of the proposed DermoGAN model, shown in Fig. 1, is to estimate a mapping $G_{A2B}$ from an RCM image domain (A) toward a binary domain (B). The mapping is learned using two connected complementary tasks. The first one learns the RCM image noise and texture model (the likelihood of the image) from two sets of unpaired images: a set of RCM images and a set of (synthetic) binary images (obtained by simulating a prior model). The second task maps Gabor-filtered RCM images (domain C), i.e., where membranes have been highlighted, into (synthetic) binary images, to learn the global geometrical structure of the epidermal tissue. The combination of the two tasks makes the overall model structure-aware, allowing us to denoise RCM images while keeping the position and integrity of the membrane.

The proposed architecture is fully unsupervised, thus circumventing the obstacle of limited labeled data. In addition, as it does not rely on training with a manually generated ground truth, as opposed to supervised approaches, such as U-net, its accuracy cannot be impacted by incorrectly labeled data, i.e., missing cells in the ground truth or wrong detections.

Each task is a cycle-GAN, made of two generators, denoted $G_{A2B}$ and $G_{B2A}$ in the first task and $G_{C2B}$ and $G_{B2C}$ in the second task, and two discriminators, denoted $D_{B_{1}}$ and $D_{A}$ in the first task and $D_{C}$ and $D_{B_{2}}$ in the second task, making a total of eight networks in the model.

#### 2.1.1 Generator and discriminator architecture

The generator and discriminator networks form pairs ($G_{A2B}/D_{B_{1}}$ and $G_{B2A}/D_{A}$, $G_{C2B}/D_{B_{2}}$, and $G_{B2C}/D_{C}$). A generator takes a 256×256 image as input, down-samples it to extract high-level features and reduce spatial resolution, applies a succession of residual blocks to these features, and then up-samples them to increase the spatial resolution backup and generate the output, as described in Fig. S1 in the Supplementary Material. Each generator aims to create realistic target images taking a source image as input. The generators are constrained by an identity loss,^19^ to ensure that the generator does not modify a target domain image if used as an input, encouraging it to be an identity mapping, i.e., $G_{A2B}(B)\approx B$. The two generators in the network should be cycle-consistent to ensure that the data are preserved during the translation process and the latter is reversible, i.e., $G_{A2B}(G_{B2A}(B))\approx B$.^20^

The weights in all generators were initiated from a Xavier (or Glorot) normal distribution^21^ such that the variation of the activations is the same across all layers to reduce the risk of the gradient exploding or vanishing and is a random number with a normal probability distribution in the range $\pm \sqrt{\frac{6}{n_{i}+n_{o}}}$, where $n_{i}=862$ is the number of input images (both real RCM images and Gabor-filtered ones) and $n_{o}=400$ is the number of output images (synthetic binary images). The weights of the generators were then updated by minimizing three loss functions (see Fig. 1).

In the case of $G_{A2B}$, these losses are as follows:

(1) adversarial loss calculated with a mean squared error (MSE) between the generator and its associated discriminator, here, $D_{B_{1}}$, such that for a pixel at coordinates [i,j] of the generated image $Gen_{B}$, it is defined as
  $$\mathrm{MSE}(D_{B_{1}}({\mathrm{Gen}}_{B}),1)=\frac{1}{n_{t}}\overset{n_{t}}{\underset{i,j}{\sum }}(D_{B_{1}}({\mathrm{Gen}}_{B})(i,j)-1{)}^{2},$$ (1)
  where $n_{t}$ is the size of the tensor outputted by the discriminator.
(2) identity loss with a mean absolute error (MAE) between the input image $I_{B}$ from domain B and the theoretical identity mapping $Id_{I_{B}}=G_{A2B}(I_{B})\approx I_{B}$, defined for image $I_{B}$ at pixel [i,j]
  $$\mathrm{MAE}(Id_{I_{B}},I_{B})=\frac{1}{n_{i}}\overset{n_{i}}{\underset{i,j}{\sum }}|Id_{I_{B}}(i,j)-I_{B}(i,j)|,$$ (2)
(3) forward or backward cycle consistency loss with an MAE between an input image $I_{B}$ from domain B and the corresponding reconstructed image ${\mathrm{Rec}}_{I_{B}}=G_{A2B}(G_{B2A}(I_{B}))$, defined at pixel of coordinates [i, j] as
  $$\mathrm{MAE}({\mathrm{Rec}}_{I_{B}},I_{B})=\frac{1}{n_{i}}\overset{n_{i}}{\underset{i,j}{\sum }}|{\mathrm{Rec}}_{I_{B}}(i,j)-\;I_{B}(i,j)|.$$ (3)

This loss function participates 10 times more in the update of the generator weights compared with the adversarial MSE loss.

The generators were trained with the adaptive moment estimation (ADAM) optimizer with an initial learning rate of 0.002 and a decay rate of the gradient exponential moving average of 1.

The discriminators take an image as input and output the classification results (real versus fake) in a tensor. Each discriminator aims to distinguish between real and generated target images, thus working against its matching adversary generator, which aims to create indiscriminable generated target images. These two networks are connected through the adversarial loss in Eq. (1),^20^ and the discriminator loss function is defined as

$$\frac{1}{2}\mathrm{MSE}(D_{B_{1}}({\mathrm{Gen}}_{B}),1)+\frac{1}{2}\mathrm{MSE}(D_{B_{1}}(I_{B}),0).$$ (4)

Training each generator/discriminator pair simultaneously allows the cycle-GAN to learn the bidirectional image-to-image translation between two unpaired domains.

#### 2.1.2 Multi-task approach

RCM images are noisy and heterogeneous due to tissue-induced scattering^22^ and are non-specific to organelles and macro-structures. This makes the identification of keratinocytes on RCM images a challenging task, whether done manually or automatically. In this case, cell identification requires two simultaneous tasks to capture the breadth of information in confocal images: noise removal and membrane identification. Multi-task learning allows for concurrent execution of these two related tasks, improving overall performance by leveraging complementary information and sharing representations.^23^ This reasoning mimics the expert’s approach to manual cell identification on RCM images, i.e., focusing on bright tube-like membranes while ignoring the bright blob-like noise.

Noise removal was performed using a first cycle-GAN, learning the translation between RCM and binary images obtained by simulating the structure of keratinocytes^8^ (described in Sec. 3.3.1), whereas membrane identification was performed by learning the mapping between binary images and Gabor-filtered RCM images, i.e., where membranes were highlighted.

The multi-task model is optimized through the soft-sharing of parameters,^24^ as the two tasks do not share any hidden layer but are connected through their loss function, as shown in Fig. 1. Indeed, at each update of the loss function, those associated with the generators creating the binary images, i.e., $G_{A2B}$ and $G_{C2B}$, are updated through their regular optimization, and then, the maximum value of the two trios of losses is set as the loss function for both generators to synchronize training across the two tasks of noise removal and membrane identification.

### 2.2 Refining the Results with Star-convex Polygons

**Fig. 2:**
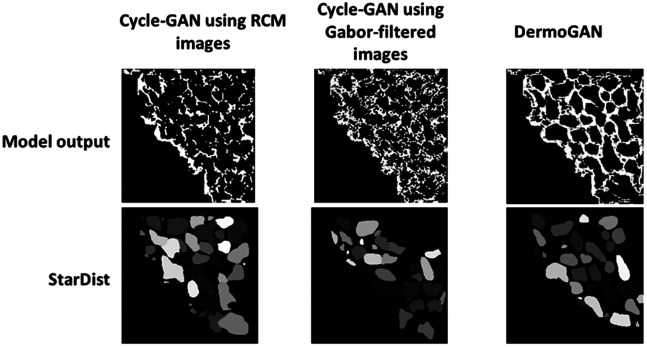
Comparison of the two cycle-GAN-based approaches and the proposed DermoGAN. DermoGAN outperforms both methods.

**Fig. 3:**
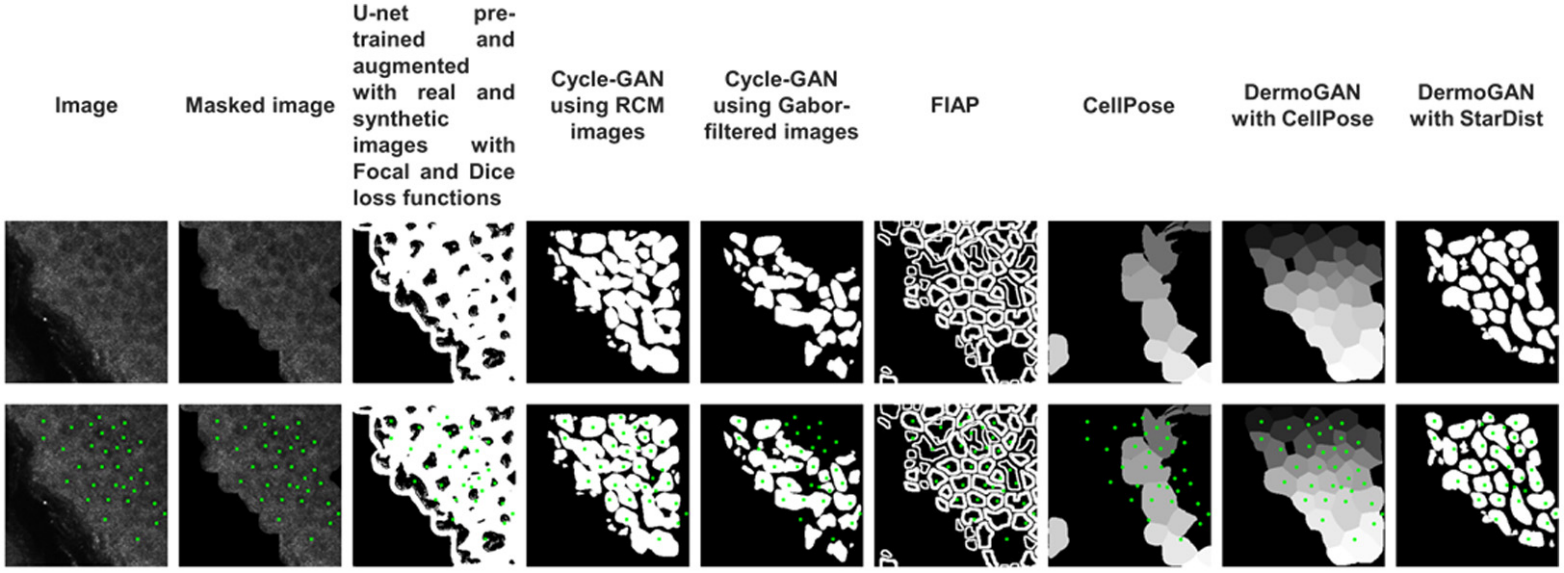
RCM image analyzed with the eight presented algorithms. DermoGAN outperforms five out of six other methods on all images and outperforms FIAP on six out of nine images.

The proposed method is a top-down approach to cell detection. The DermoGAN roughly localizes individual cell locations, but post-processing is required, as shown in Figs. 2 and 3. Indeed, applying the obtained mapping $G_{A2B}$ to a locally normalized RCM image results in an incomplete binary image. To guarantee that the outside contour of tissue where the keratinocytes are detected is closed, we compute the alpha shape^25^ of the incomplete binary mask at a set level of refinement, such that the tissue comprises only one volume per external contour and is not broken down into smaller shapes and that the alpha-shape contour matches the actual tissue area. Small holes in the membrane are then closed using a connected components analysis.^26^

We assume that all cells are star-convex shapes. However, non-star-convex polygons can result from the false merging of two or more cells due to the lack of contrast on the membranes. To split these shapes, we use the pre-trained convolutional neural network StarDist^17^ to detect star-convex polygons within the contours detected by the DermoGAN model, consequently refining our results, countering any missed cells, and reducing the number of false negatives, as shown in Fig. 2.

## 3 Experiments and Results

### 3.1 Dataset

RCM images were captured using a Vivascope 1500 (Lucid, Inc., Rochester, New York, United States) reflectance confocal microscope, on the volar forearm of 60 children (3 months to 10 years) and 20 adult women (25 to 40 years) and on the volar forearm and cheek of 80 other adult women (40 to 80 years). All participants have Fitzpatrick types between I and III, were in good health, and had no history of skin disease. The study was initiated following approval from an independent institutional review board and in accordance with the Declaration of Helsinki (studies 19.0198 and 20.0022). Subjects or their guardian gave written informed consent prior to study initiation.

The image size was 1000×1000  pixels, corresponding to $500\times 500\;\mu m^{2}$, with a resolution of $1\;\mu m^{2}$ per pixel. A region of interest (ROI) mask was generated for and applied to all images used across all six tested methods. The ROI was identified by distinguishing the tissue from the dark background, due to the skin micro-relief lines, using a morphological-geodesic-active-contour, and removing non-informative areas in the tissue, due to low contrast and a drop in the signal-to-noise ratio, through a texture classification with a support vector machine on four features of the gray level co-occurrence matrix (homogeneity, contrast, dissimilarity, and energy^8^).

Images used in DermoGAN, U-net, and both approaches using a cycle-GAN were of the size 256×256  pixels and obtained by splitting the full image into nine non-overlapping square patches of 256×256  pixels. The full-image analysis pipeline network used full RCM images.^10^

DermoGAN and both cycle-GAN models were trained using the same 862 RCM images of the size 256×256  pixels and 400 synthetic images. The number of synthetic images used in the training of the models was determined empirically. Indeed, we noticed that adding more images did not improve performance but increased computational time. Using 400 images was the right balance between performance and computational time and power. The RCM images represent both the volar forearm and cheek and include participants ages 0 to 80 years.

Image classification in one of the four epidermal layers was obtained using a hybrid deep learning algorithm,^27^ allowing to focus only on images of the *stratum granulosum* (SG) and *stratum spinosum* (SS), where keratinocytes are visible and identifiable on RCM images and arranged in a honeycomb pattern^28^ inside of islands surrounded by dark grooves representing micro-relief lines.^8^^,^^29^

Ground truth used for the evaluation of all the tested approaches was generated by a single expert manually pointing out cell centers on nine RCM images of seven subjects (children and adult women), ages 5 months to 35 years old. The use of ground truth generated by a sole expert in the evaluation of our results is a limitation of our work but is justified by the previously documented inter-expert variation in keratinocyte identification.^8^

#### 3.1.1 Generated binary synthetic images

**Fig. 4:**
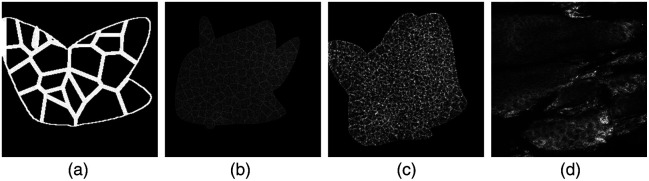
Synthetic images used in the training of the models. (a) A synthetic binary image used in the DermoGAN training. (b) and (c) RCM images of different noise levels and cell sizes used in U-net training. (d) Real RCM image. RCM, reflectance confocal microscopy.

Binary (synthetic) images, of the size 256×256  pixels, were created by generating a random tissue mask using random Bezier curves. Within these shapes, seeds, mimicking cell centers, were used to initiate a Voronoi tessellation, which have been previously used to represent both skin cells^30^ and other types of cells.^31^^–^^33^ These synthetic binary images are user-controlled, and the associated ground truth is given by the seed locations. The seeds are generated with a “hard-core” process simulation controlled by the density parameter (see Fig. 4).

Synthetic RCM images were generated by adding different levels of noise and brightness heterogeneity to the binary images, as shown in Fig. 4 and detailed in Ref. 8.

### 3.2 DermoGAN Implementation Details

The used model was trained for 5172 epochs on 46.9 central processing unit (CPU) cores and 85.4 GiB of random access memory (RAM). The training took ∼4 days. All deep learning models were implemented using PyTorch.

Intermediate models were saved every 400 epochs, and the model with the best performance, i.e., accuracy metrics (precision and recall summarized into the F1-score), was chosen.

### 3.3 Comparison to Other Automated Methods

The proposed method is compared with seven other approaches: (1) a deep learning approach based on the U-net architecture,^16^ (2) a StarDist algorithm applied to Gabor-filtered RCM images, (3) a cycle-GAN trained to translate RCM images into binary images, (4) a second cycle-GAN trained to turn Gabor-filtered RCM images into binary images, (5) a full-image analysis pipeline based on traditional image analysis methods,^8^^,^^10^^,^^34^ (6) CellPose algorithm for cellular segmentation, and (7) DermoGAN followed by postprocessing with CellPose.

The proposed combination of cycle-GAN models into a multi-task approach improves results by mimicking manual expertise, disregarding noise to focus on membrane location and tissue structure.

#### 3.3.1 U-net

A U-net architecture, pre-trained on the 2012 ImageNet Large Scale Visual Recognition Challenge dataset^35^ with an EfficientNetB3 backbone,^36^ was further trained on 43 real RCM images of the size 256×256  pixels (four participants, 20 to 35 years) and 203 synthetic RCM images and tested on 13 real RCM images and 68 synthetic RCM images. The corresponding ground truths were obtained by the same expert and do not include the nine images used in the model evaluation. The network used a combination of two loss functions: Dice loss^37^ and focal loss^38^ to account for class imbalance between cell membranes and background. The model is trained with the ADAM optimizer with an initial learning rate of 0.0001, a batch size of 64, and a sigmoid activation function. The model was trained for 500 epochs on 46.9 CPU cores and 85.4 GiB of RAM. Training took ∼3 days. The selected U-net model was obtained following multiple iterations, as described in Refs. 8 and 39.

#### 3.3.2 StarDist applied to Gabor-filtered images

A Gabor filter was applied to ROI-masked RCM images to highlight membrane positions. The result was normalized with a histogram equalization and binarized with a Gaussian adaptive thresholding. A pre-trained StarDist was then applied to the binary-masked Gabor-filtered RCM image.

#### 3.3.3 Cycle-GAN-based models

Two cycle-GAN models were trained on 862 RCM images and 400 binary images, each one representing a task in the DermoGAN architecture, to evaluate each model independently and later emphasize the importance of combining the two tasks into one architecture. The first one aimed to translate RCM images into binary images, whereas the second sought to convert Gabor-filtered RCM images into binary images. Both tested cycle-GAN models were refined using star-convex shape detection as performed in the DermoGAN architecture. Training was performed for 12,068 epochs on 46.9 CPU cores and 85.4 GiB of RAM and took 2 days.

#### 3.3.4 Full image analysis pipeline (FIAP)

A three-step pipeline for keratinocyte detection^8^^,^^10^^,^^34^ based on membrane detection using image filters was applied to full RCM images of size 1000×1000  pixels. First, the ROI containing the epidermal cells is identified. Texture filters (Gabor and Sato filters) are then applied to the image to accentuate tube-like structures (membranes) within the ROI and identify individual cells within it. The detected contours are then post-processed using prior biological knowledge^40^ on expected cell size to remove contours that are too small (cell area<100  pixels for contours detected on RCM images of the SG and cell area<50  pixels for contours detected on RCM images of the SS). The texture filters were reapplied locally on detected regions presumed to be too big to be considered a single cell and divided into multiple cells if needed. The FIAP is applicable to images of the SG and SS and requires a different set of parameters for each layer, which were determined manually. Computational time is 7 to 10 min per image depending on image complexity and required post-processing steps.

#### 3.3.5 CellPose

The pre-trained CellPose model was applied, without retraining, to the full RCM images of the size 1000×1000  pixels where the ROI had already been identified. CellPose is a generalist single-class instance segmentation algorithm optimized for cellular segmentation across different microscopy modalities. The model was run with the following parameters: ROI diameter = 50 for SG images, ROI diameter = 50 for SS images, and flow threshold = 0.4, cell probability threshold = 0.2, and stitch threshold = 0.

#### 3.3.6 DermoGAN followed by CellPose

We replaced the previously described postprocessing step with StarDist using the TissueNet cell model available in CellPose. The model was run with the following parameters: ROI diameter = 50 for SG images, ROI diameter = 50 for SS images, and flow threshold = 0.4, cell probability threshold = 0.2, and stitch threshold = 0.

All eight tested methods were evaluated against the same RCM images. While the two cycle-GAN-based approaches and DermoGAN were trained on the same images, U-net was not. Indeed, U-net is a supervised learning approach, and ground truth was not available for all images used in the training of the other tested methods. This may limit the comparability of the approaches to each other but highlights the importance of unsupervised learning methods, which are not limited by the available labeled data.

### 3.4 Keratinocyte Identification Results

The proposed DermoGAN architecture was evaluated using nine full RCM images, each divided into nine patches. Accuracy (precision and recall summarized into the F1-score) was calculated using d-accuracy^41^ against a manually obtained ground truth and compared with the results obtained using the eight described methods, as shown in Fig. 3 and Table 1.

**Table 1:** Comparison of median F1-score (computed with d-accuracy^41^) for all eight tested approaches.

	U-net-based architecture	StarDist on Gabor-filtered images	Cycle-GAN using RCM images	Cycle-GAN using Gabor-filtered images	FIAP	CellPose	DermoGAN with CellPose	DermoGAN with StarDist
Training data size	Training: 43 real RCM images and 203 synthetic RCM imagesTesting: 13 real RCM images and 68 synthetic RCM images.	No training required	862 RCM images and 400 binary images	862 RCM images and 400 binary images	No training required	No training required	862 RCM images and 400 binary images (no training required for CellPose)	862 RCM images and 400 binary images (no training required for StarDist)
Median F1-score	47.9	38.9	41.6	30.4	65.1	35.0	62.4	69
Standard deviation	12.2	12.1	6.4	8.3	7.4	16.5	8.7	4.2

The poor performance of the pre-trained U-net model augmented with real and synthetic RCM images with Focal and Dice loss functions is in part due to the limited training set. Being a supervised approach, it may also suffer from missing cells in the ground truth used for training due to inter and intra-expert variability and subjectivity in manual keratinocyte identification on RCM image^8^ and from membranes in the ground truth images created by Voronoi tessellation initiated from manually determined cell centers, not matching the actual membrane position in RCM images.

The pre-trained StarDist applied to Gabor-filtered images also performs poorly. Indeed, although the Gabor filter highlights most membranes, it may also highlight noise due to, for example, organelles, leading to false positives and low precision. Although the StarDist post-processing greatly improves results by segmenting (correctly or not) the detected contours into star-convex shapes, it does not manage to correct for all missing cells, leading to false negatives and consequently low recall and overall low F1-score.

Both cycle-GAN-based approaches have low F1-scores as they fail to detect complete membranes, as shown in Fig. 2. Indeed, the cycle-GAN model trained on RCM and binary images struggles to distinguish between noise and microstructures making up the membranes. On the other hand, the cycle-GAN trained on Gabor-filtered images with binary images is corrupted by the spatial correlation of noise and fails to detect any structure present in the image, as seen in Fig. 2, which also hints at the reason behind the DermoGAN greater performance. Indeed, it seems that adding up the two independent cycle-GAN outputs would close most holes in the detected membranes by focusing on membrane detection and omitting any noise visible in them.

The pre-trained CellPose applied to the full RCM images without retraining has a low F1-score and better performance for SG images compared with SS images, as shown in Table S1 in the Supplementary Material. Overall precision is higher than recall, showing that this method is more conservative in detecting positive instances.

Overall, the last test method using the TissueNet cell model from CellPose as a postprocessing step to the proposed DermoGAN model performs well against both SG and SS images. This method has a good F1-score but tends to have higher precision than recall. It is still outperformed by the selected method (DermoGAN followed by StarDist-based postprocessing), which has a better trade-off between recall and precision and therefore is better suited for keratinocyte identification on RCM images.

Both DermoGAN and FIAP outperform the other models, as shown in Table 1 and Tables S1 and S2 in the Supplementary Material, and show a great trade-off between precision and recall. DermoGAN has a higher F1-score than FIAP for six out of nine images. The first seems to favor recall and is less likely to miss existing cells and produce false negatives, whereas the second seems to favor precision and is less likely to invent cells and create false-positive detections.

The DermoGAN architecture does not require manual parametrization nor a different set of parameters per epidermal layer, contrary to the FIAP. This argues in favor of the DermoGAN since multiple epidermal layers are often present in one RCM image. Once trained, its execution time is faster. It is based on the discovery of potentially unknown patterns in the image, making it less explainable than the FIAP. The latter is built on membrane detection using tubeness filters, with all its parameters being determined using general prior knowledge of the morphological features of the studied tissue. It is well documented that the keratinocyte area increases with age and differs from one body site to another; thus, general parameters determined on a specific dataset may not be appropriate for all images. This point favors the DermoGAN architecture as more adaptable to different datasets and potentially to different image acquisition techniques and/or observed tissue.

## 4 Discussion

Although the presented method was trained using RCM images for the detection of keratinocytes, we hypothesized that it can be extended without retraining to images generated by other instruments. Indeed, multi-task learning methods tend to perform well on domain adaptation and generalization and are therefore less data-dependent. On the other hand, such adaptability can lower pixel-level segmentation and therefore is more suited when the accuracy is calculated at the object level and not at the pixel level. In the following paragraphs, we aim to explore the potential applications of DermoGAN to images acquired using different imaging modalities. Accuracy metrics will not be presented as this is a preliminary step to future research.

**Fig. 5:**
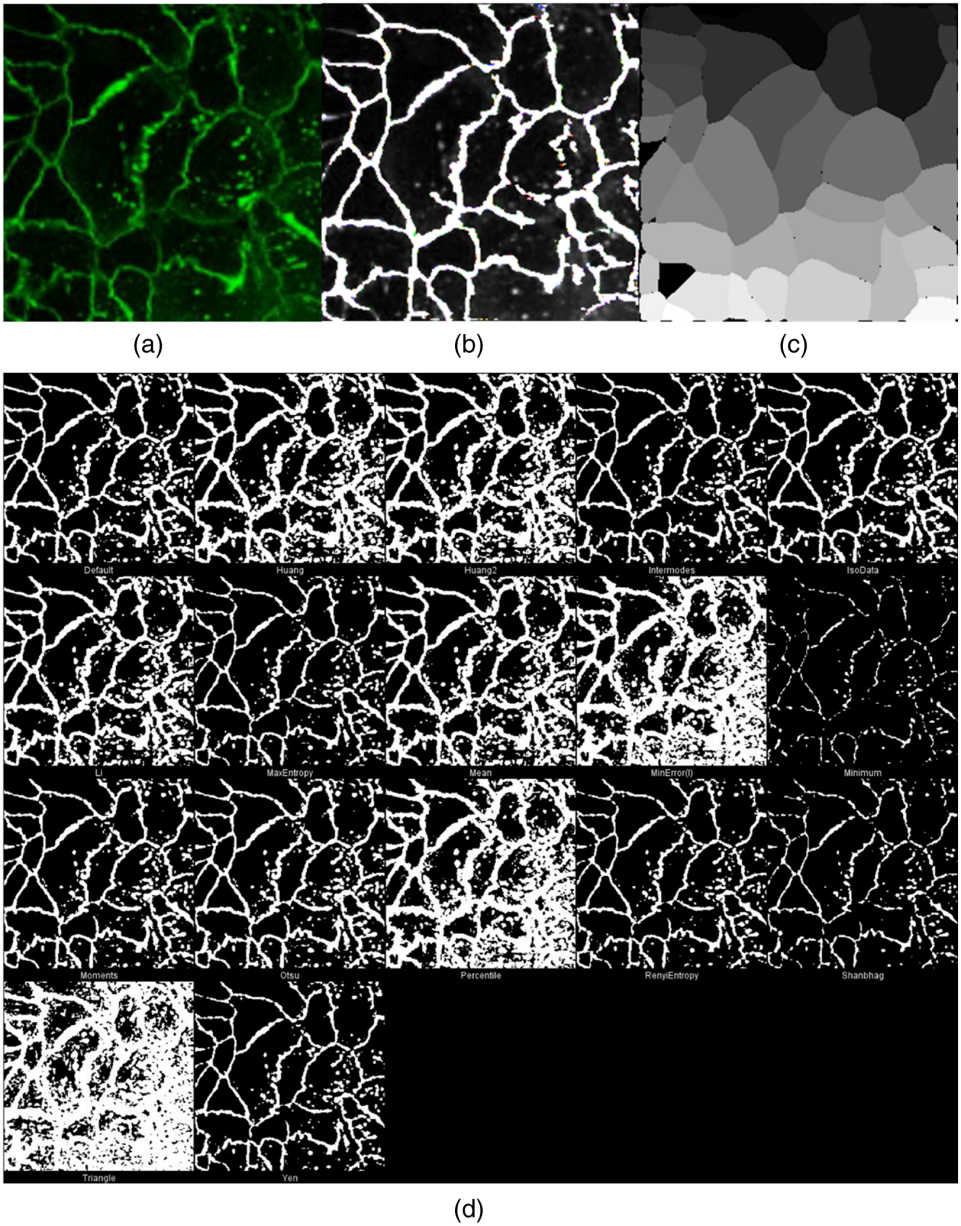
DermoGAN can be extended to images acquired by different imaging techniques using retraining and outperforms traditional thresholding algorithms. (a) Input fluorescence microscopy images. (b) Output of DermoGAN applied to image A. (c) Output of CellPose cyto model. (d) Application of 18 thresholding approaches to the same image.

To explore the possibility of applying DermoGAN to other images without retraining, we applied the presented model trained on RCM images to fluorescence microscopy images and compared the obtained results with 18 thresholding methods and the pre-trained CellPose cyto model, as shown in Fig. 5. We observed that although trained on different images of a different tissue, DermoGAN managed to identify membranes while omitting the noisy background and outperformed traditional thresholding methods. It had a similar performance to the pre-trained CellPose model which has better performance at image borders but misses a cell. It is important to note that the fluorescence microscopy image does show a similar tissue organization, i.e., cohesive tissue with cells *sharing* membranes, to RCM images of the epidermis. However, when tested on cell culture images where cells were not always confluent, we noticed a loss in accuracy when using DermoGAN for cell identification. We therefore trained a second model on images where cells were not confluent using a different tissue prior to simulating binary images and a different filter to enhance contours, which will be referred to for simplicity as DermoGAN2.

### 4.1 Retraining the Model with Only Synthetic Images

DermoGAN2 was trained entirely on synthetic images. This served as a test of the generalization of the method when the available dataset is even more limited and serves to prove that the combination of the two tasks in the proposed model can capture general information and therefore can be extended to different images and tissues with similar organization, architecture, or texture, even when the images of interest were not included in the training set.

**Fig. 6:**
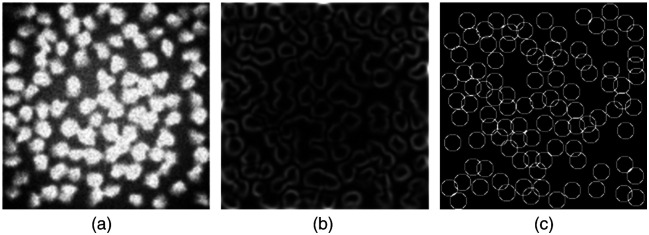
DermoGAN2 was trained entirely on synthetic images. (a) Synthetic non-confluent images created using the SIMCEP. (b) Canny-filtered non-confluent images created using the SIMCEP. (c) Binary non-confluent images.

The first task in DermoGAN2 maps synthetic non-confluent images created using SIMCEP software for the simulation of fluorescence microscope images of cell populations^42^ [Fig. 6(a)] to binary non-confluent images [Fig. 6(c)]. The binary images were obtained by simulating a marked point process embedding a constraint on the overlap between objects defined by disks.^43^ The second task aims to learn the translation of Canny-filtered synthetic non-confluent images [Fig. 6(b)] toward the same binary non-confluent images.

The resulting DermoGAN2 was then applied to images of cell cultures and mass spectroscopy images.

#### 4.1.1 DermoGAN2 on cell culture images

**Fig. 7:**
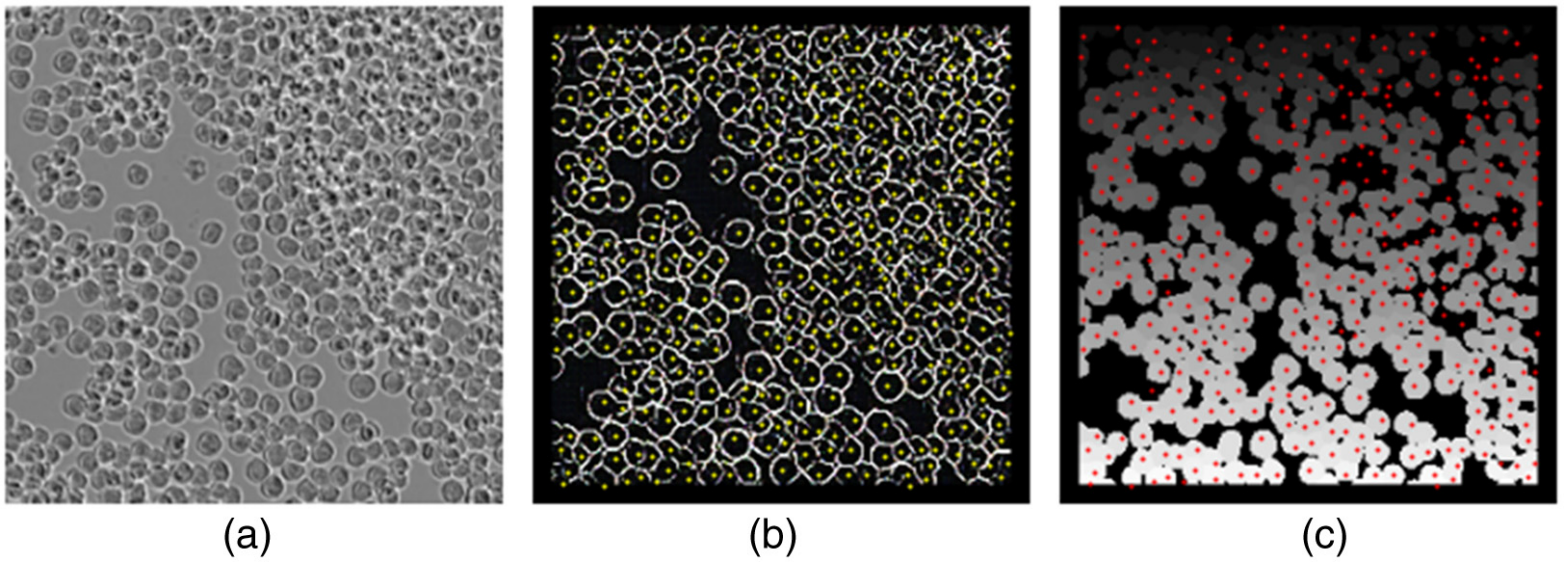
DermoGAN2 applied to an image of confluent BV-2 cells (a), resulted in the accurate detection of cells with both DermoGAN (b) and CellPose (c). Manually determined cell centers were plotted on the DermoGAN2 output in yellow and on the CellPose output in red.

We applied DermoGAN2 on an image of BV-2 microglial cells derived from C57/BL6 murine from the LIVECell dataset,^44^ as seen in Fig. 7. We obtained an accurate segmentation of the cells on the image. To avoid border effects in the image, a 10px frame was applied to the image. We compare DermoGAN output to the pre-trained CellPose cyto model (*diameter = 10*, *flow threshold = 0.4*, *cell probability threshold = 0*, and *stitch threshold = 0*). We observe a drop in performance for both methods.

**Fig. 8:**
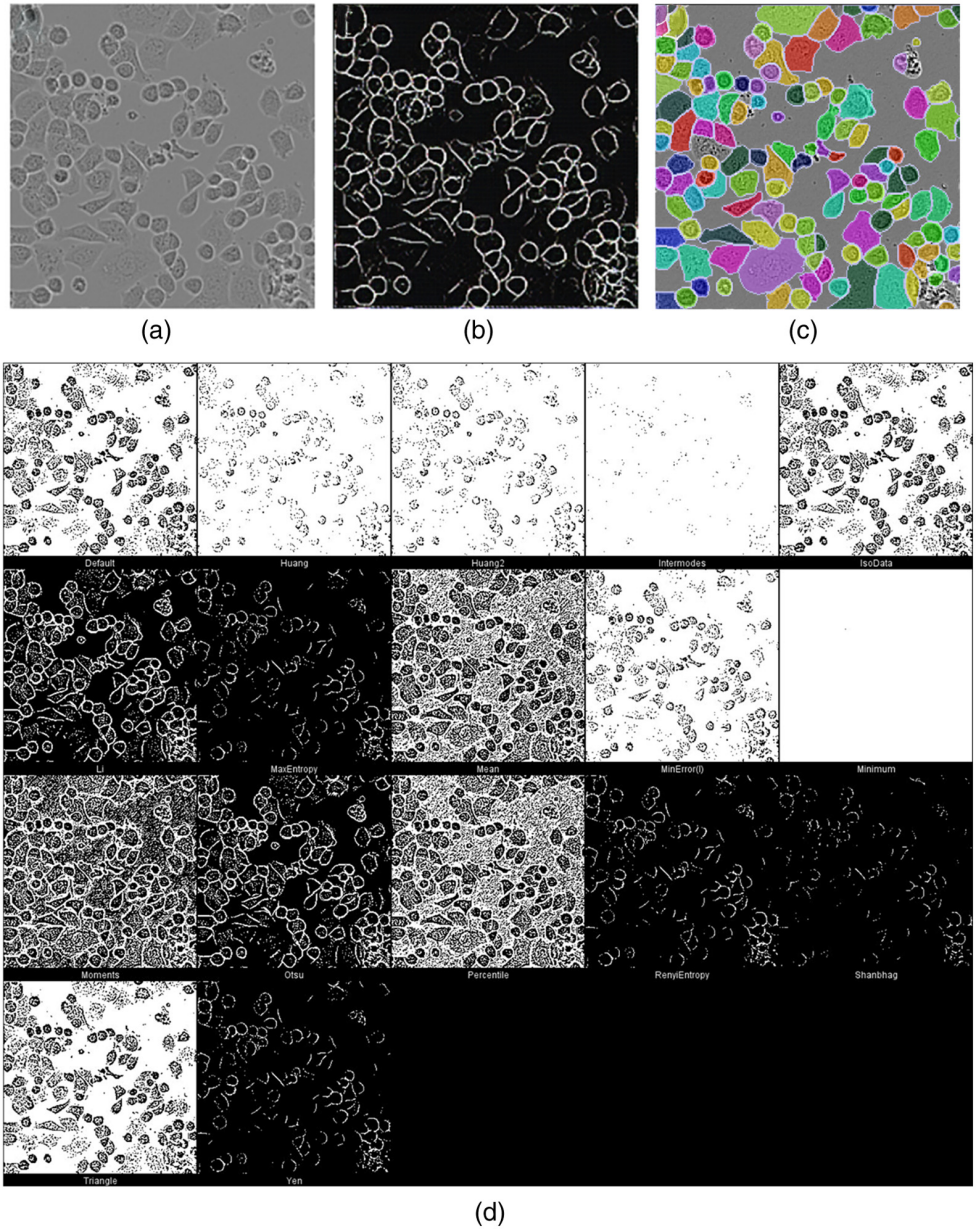
DermoGAN2 applied to an image of SK-BR-3 cells (a) resulted in accurate detection with both DermoGAN (b) of cells and CellPose (c), compared with thresholding methods (d).

We also applied DermoGAN2 to an image of the SK-BR-3 human breast cancer cell line, where cells display morphological heterogeneity [Fig. 8(a)]. Good cell detection was observed [Fig. 8(b)] on most cells when contrast is high enough. This proves that DermoGAN2 can be extended to different cell shapes and is not limited to the detection of the circular cells it was trained on and that it is not restricted by the aspect of the cells. Indeed, the synthetic images used for training the model were more similar to fluorescence images, with high luminosity, which is not the case for the tested cell culture images. Similar results can be observed when applying the pre-trained CellPose live-cell model (*diameter = 10*, *flow threshold = 0.4*, *cell probability threshold = 0*, and *stitch threshold = 0*) [Fig. 8(d)], which seems to visually outperform DermoGAN in this case.

#### 4.1.2 DermoGAN2 on mass spectroscopy images

**Fig. 9:**
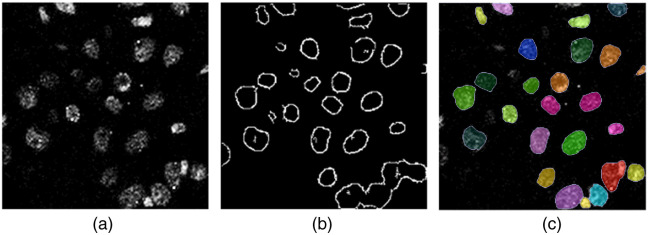
DermoGAN2 applied to a mass spectroscopy image (a) resulted in accurate cell detection with both DermoGAN (b) with the merging of adjacent cells and CellPose (c).

Similar observations were made on mass spectroscopy images, where DermoGAN2 managed to detect hazy cell contours, with a tendency to merge close cells into one detected region, as shown in Fig. 9. This can be solved by post-processing using StarDist as done on RCM images. When applying the pre-trained CellPose cyto model to the same mass spectroscopy image, we observe a drop in detection in areas suffering from a drop in contrast, which is not the case for DermoGAN, but borders are sharper when using CellPose compared with DermoGAN.

The success of DermoGAN in segmenting cohesive tissues, including that of DermoGAN2 in detecting non-confluent cells, highlights the importance of the binary denoised image domain (domain B in DermoGAN). This domain serves as a prior domain incorporating anterior knowledge in the model by describing the structure of the studied tissue. In DermoGAN, the prior is represented as a tissue island containing adjacent cells of similar size, while in DermoGAN2, this prior is represented by circular non-confluent cells. This prior domain summarizes our degree of certainty concerning the studied tissue and steers the training of the model toward the right solution. Therefore, to obtain the best results, the appearance of the tissue should guide the choice of the appropriate DermoGAN model based on the corresponding prior domain.

In all the iterations of DermoGAN so far, training was performed using cell membrane positions. It would be of interest to see if the performance of the models could be improved by replacing the images representing cell membranes in the training with images representing the entire cell (cell membrane and inside of the cell), therefore having two classes in the data, cell versus background instead of cell membrane versus background as currently done.

## 5 Conclusion

This paper has presented a novel multi-task cycle-GAN architecture for the identification of keratinocytes on RCM images and was compared with seven other methods. Supervised deep learning approaches obtained poor scores due to the lack of annotated data, even when using transfer learning. Unsupervised learning, such as cycle-GAN, failed to capture information at different scales simultaneously. Therefore, the FIAP approach outperformed these attempts. However, the proposed DermoGAN, which combines two cycle-GANs to embed both local and global structure information, outperformed the classical FIAP, in terms of accuracy and execution time.

We showed that the proposed fully unsupervised architecture used with or without retraining on other types of imaging and tissue types, bypassing the problem of required annotated data and potential label noise/missing labels, provided the creation of simulated data and that it is not limited by the training set but rather determined by the prior data domain, i.e., tissue organization and architecture in the training set. It would be of interest to generate new prior domains using marked point processes to generate more specific priors, which would help extend the use of DermoGAN to images with multiple cell types or when the spatial dependence among different structures is important.

## Supplementary Material



## Data Availability

The data and code that support the findings of this study are available upon reasonable request from the corresponding author, I. L.
