# Supplementary material for: DermoGAN: multi-task cycle generative adversarial networks for unsupervised automatic cell identification on in-vivo reflectance confocal microscopy images of the human epidermis
Source: J Biomed Opt. 2024 Aug 2;29(8):086003. doi: 10.1117/1.JBO.29.8.086003 (PMC11294601; doi:10.1117/1.JBO.29.8.086003)
Supplement: Supplementary file 1 [file JBO_029_086003_SD001.docx]

**
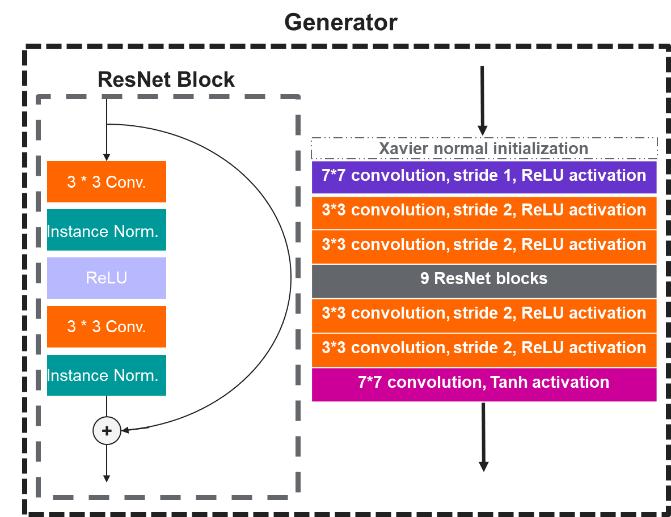
**

**
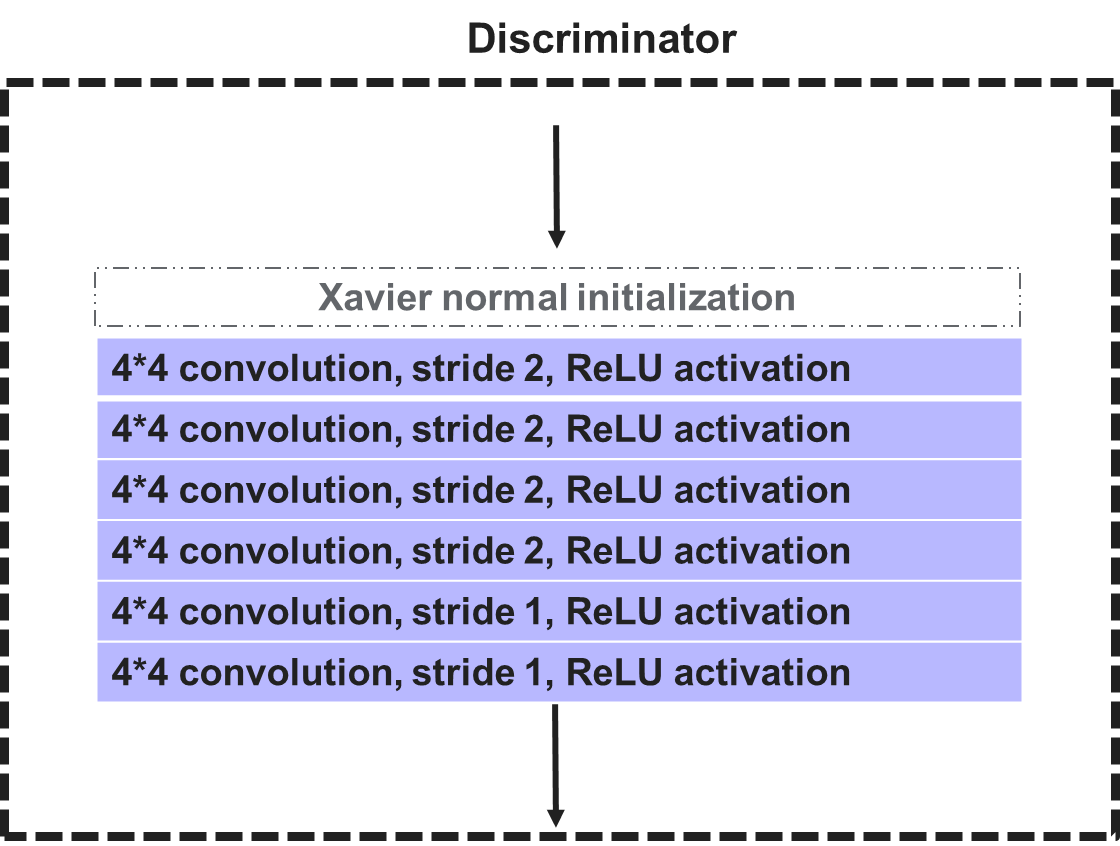
**

**Fig. S1** Structure of the generator and discriminator networks used in the cycle-GAN and DermoGAN approaches.


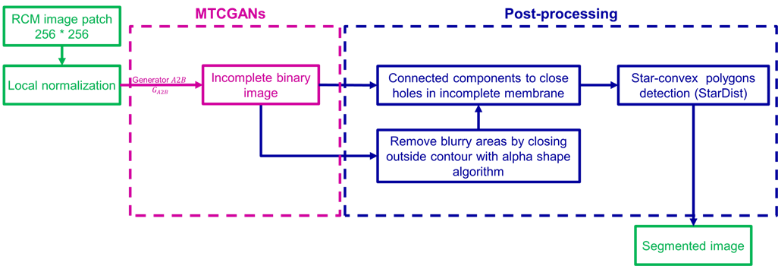


**Fig. S2** To obtain keratinocytes positions. We apply the $G_{A2B}: A\to B$ network to locally normalized RCM image and obtain an incomplete cell identification, which is then cleaned by closing any holes in the detected membrane and the outside contour, and finally the cell identification is refined using StarDist algorithm. RCM, reflectance confocal microscopy.

**Table S1** Comparison of all accuracy metrics for all eight tested approaches (in %).

| Image | Layer | U-net pre-trained and augmented with real and synthetic images with Focal and Dice loss functions | | | StarDist on Gabor-filtered images | | | Cycle-GAN using RCM images | | | Cycle-GAN using Gabor-filtered images | | | FIAP | | | CellPose | | | DermoGAN + CellPose | | | DermoGAN + StarDist | | |
| --- | --- | --- | --- | --- | --- | --- | --- | --- | --- | --- | --- | --- | --- | --- | --- | --- | --- | --- | --- | --- | --- | --- | --- | --- | --- |
|  |  | Precision | Recall | F1-score | Precision | Recall | F1-score | Precision | Recall | F1-score | Precision | Recall | F1-score | Precision | Recall | F1-score | Precision | Recall | F1-score | Precision | Recall | F1-score | Precision | Recall | F1-score |
| 1 | SS | 50.3 | 29.8 | 37.5 | 37.1 | 47.5 | 41.6 | 47.8 | 46.8 | 47.3 | 42.9 | 40.8 | 41.8 | 46.2 | 89.4 | 61.0 | 31.3 | 14.1 | 19.5 | 83.6 | 61.5 | 70.9 | 68.4 | 73.6 | 70.9 |
| 2 | SS | 37.4 | 24.6 | 29.7 | 50.3 | 53.8 | 51.9 | 40.6 | 51.7 | 45.5 | 57.7 | 33.9 | 42.7 | 52.0 | 76.1 | 61.8 | 24.2 | 12.5 | 16.5 | 75.9 | 38.0 | 50.6 | 77.9 | 51.0 | 61.6 |
| 3 | SS | 40.8 | 24.6 | 30.7 | 50.5 | 44.4 | 47.3 | 45.9 | 42.6 | 44.2 | 50.4 | 26.7 | 34.9 | 48.9 | 79.6 | 60.6 | 25.2 | 11.0 | 15.3 | 65.8 | 40.3 | 50.0 | 72.5 | 60.7 | 66.1 |
| 3 | SG | 44.8 | 51.3 | 47.9 | 43.8 | 76.1 | 55.6 | 31.9 | 65.2 | 42.9 | 30.6 | 41.3 | 35.2 | 63.9 | 60.5 | 62.2 | 35.8 | 38.2 | 36.9 | 65.3 | 80.3 | 72.1 | 64.9 | 82.0 | 72.5 |
| 3 | SG | 28.8 | 56.8 | 38.2 | 27.7 | 65.5 | 38.9 | 23.1 | 67.3 | 34.4 | 17.5 | 32.7 | 22.8 | 56.4 | 77.0 | 65.1 | 31.9 | 48.6 | 38.5 | 61.6 | 60.8 | 61.2 | 53.4 | 85.1 | 65.6 |
| 3 | SG | 45.3 | 64.3 | 53.2 | 14.4 | 72.0 | 24.0 | 29.9 | 68.4 | 41.6 | 22.2 | 28.0 | 24.8 | 71.3 | 81.8 | 76.2 | 45.3 | 27.3 | 34.1 | 56.0 | 70.5 | 62.4 | 53.2 | 84.8 | 65.4 |
| 3 | SG | 42.4 | 70.7 | 53.0 | 14.7 | 78.7 | 24.7 | 19.7 | 58.7 | 29.5 | 16.8 | 31.8 | 22.0 | 71.6 | 90.7 | 80.0 | 52.3 | 60.0 | 55.9 | 62.0 | 81.7 | 70.5 | 63.9 | 92.0 | 75.4 |
| 3 | SG | 60.5 | 55.3 | 57.8 | 23.0 | 62.8 | 33.7 | 30.1 | 43.6 | 35.7 | 34.4 | 27.3 | 30.4 | 78.9 | 56.3 | 65.7 | 56.1 | 25.4 | 35.0 | 71.7 | 45.8 | 55.9 | 65.8 | 75.8 | 70.4 |
| 3 | SG | 50.5 | 86.6 | 63.8 | 14.8 | 79.5 | 25.0 | 21.0 | 70.9 | 32.4 | 18.8 | 26.1 | 21.8 | 64.1 | 87.4 | 74.0 | 51.5 | 78.7 | 62.3 | 58.8 | 82.3 | 68.6 | 57.4 | 86.6 | 69.0 |

**Table S2** Comparison of median accuracy metrics for all eight tested approaches (in %).

|  | | U-net pre-trained and augmented with real and synthetic images with Focal and Dice loss functions | | | StarDist on Gabor-filtered images | | | Cycle-GAN using RCM images | | | Cycle-GAN using Gabor-filtered images | | | FIAP | | | CellPose | | | DermoGAN + CellPose based postprocessing | | | DermoGAN + StarDist based postprocessing | | |
| --- | --- | --- | --- | --- | --- | --- | --- | --- | --- | --- | --- | --- | --- | --- | --- | --- | --- | --- | --- | --- | --- | --- | --- | --- | --- |
|  |  | Precision | Recall | F1-score | Precision | Recall | F1-score | Precision | Recall | F1-score | Precision | Recall | F1-score | Precision | Recall | F1-score | Precision | Recall | F1-score | Precision | Recall | F1-score | Precision | Recall | F1-score |
| All | Median | 44.8 | 55.3 | 47.9 | 27.7 | 65.5 | 38.9 | 30.1 | 58.7 | 41.6 | 30.6 | 31.8 | 30.4 | 63.9 | 79.6 | 65.1 | 35.8 | 27.3 | 35.0 | 65.3 | 61.5 | 62.4 | 64.9 | 82.0 | 69.0 |
|  | SD | 8.9 | 21.6 | 12.2 | 15.1 | 13.4 | 12.1 | 10.5 | 11.3 | 6.4 | 15.2 | 5.8 | 8.4 | 11.3 | 12.1 | 7.4 | 12.2 | 23.5 | 16.5 | 8.8 | 17.7 | 8.7 | 8.4 | 13.3 | 4.2 |
| SS | Median | 40.8 | 24.6 | 30.7 | 50.3 | 47.5 | 47.3 | 45.9 | 46.8 | 45.5 | 50.4 | 33.9 | 41.8 | 48.9 | 79.6 | 61.0 | 25.2 | 12.5 | 16.5 | 75.9 | 40.3 | 50.6 | 72.5 | 60.7 | 66.1 |
|  | SD | 6.7 | 3.0 | 4.2 | 7.7 | 4.7 | 5.2 | 3.7 | 4.6 | 1.5 | 7.4 | 7.1 | 4.3 | 2.9 | 6.9 | 0.6 | 3.8 | 1.6 | 2.1 | 8.9 | 13.0 | 11.9 | 4.8 | 11.4 | 4.6 |
| SG | Median | 45.1 | 60.5 | 53.1 | 18.9 | 74.1 | 29.3 | 26.5 | 66.2 | 35.0 | 20.5 | 29.9 | 23.8 | 67.7 | 79.4 | 69.9 | 48.4 | 43.4 | 37.7 | 61.8 | 75.4 | 65.5 | 60.6 | 85.0 | 69.7 |
|  | SD | 10.4 | 13.0 | 8.7 | 11.5 | 7.0 | 12.3 | 5.3 | 10.1 | 5.2 | 7.4 | 5.6 | 5.5 | 7.9 | 14.2 | 7.2 | 9.8 | 20.5 | 12.1 | 5.5 | 14.6 | 6.3 | 5.8 | 5.4 | 3.9 |
